# Multicomponent Reaction-Assisted Drug Discovery: A Time- and Cost-Effective Green Approach Speeding Up Identification and Optimization of Anticancer Drugs

**DOI:** 10.3390/ijms24076581

**Published:** 2023-04-01

**Authors:** Giovanni Graziano, Angela Stefanachi, Marialessandra Contino, Rubén Prieto-Díaz, Alessia Ligresti, Poulami Kumar, Antonio Scilimati, Eddy Sotelo, Francesco Leonetti

**Affiliations:** 1 Department of Pharmacy—Pharmaceutical Sciences, University of Bari “Aldo Moro”, 70125 Bari, Italy; 2 Center for Research in Biological Chemistry and Molecular Materials (CiQUS), Department of Organic Chemistry, University of Santiago de Compostela, 15782 Santiago de Compostela, Spain; 3 Institute of Biomolecular Chemistry, National Research Council of Italy, 80078 Pozzuoli, Italy

**Keywords:** multicomponent reactions, anticancer drugs, drug discovery, cancer

## Abstract

Multicomponent reactions (MCRs) have emerged as a powerful strategy in synthetic organic chemistry due to their widespread applications in drug discovery and development. MCRs are flexible transformations in which three or more substrates react to form structurally complex products with high atomic efficiency. They are being increasingly appreciated as a highly exploratory and evolutionary tool by the medicinal chemistry community, opening the door to more sustainable, cost-effective and rapid synthesis of biologically active molecules. In recent years, MCR-based synthetic strategies have found extensive application in the field of drug discovery, and several anticancer drugs have been synthesized through MCRs. In this review, we present an overview of representative and recent literature examples documenting different approaches and applications of MCRs in the development of new anticancer drugs.

## 1. Introduction: Multicomponent Reactions

**Figure 1:**
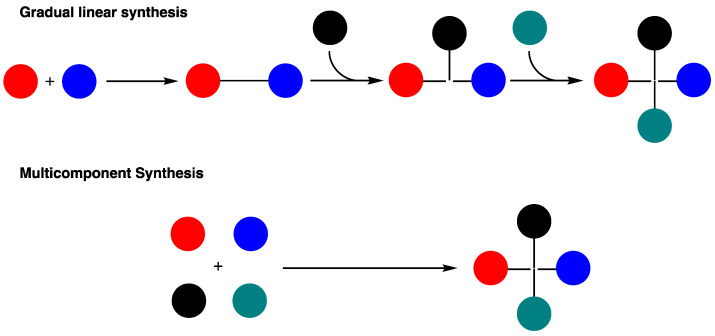
Traditional linear synthesis (3 steps) vs. multicomponent assembly (1 step).

Multicomponent reactions (MCRs) are one-pot processes in which at least three reagents are combined to assemble a novel complex product [1] with remarkable atom economy [2,3,4,5], in good to excellent yields, while saving resources such as time and energy [6,7]. MCRs provide a quicker and more efficient way to prepare chemical libraries than the methods used in traditional organic synthesis (Figure 1), offering great possibilities for molecular diversity and complexity in fewer steps and less time.

MCRs are also gaining great interest because they are generally eco-friendly and sustainable transformations [8]. Indeed, MCRs have strategic advantages over linear synthesis, being particularly appreciated by its superior atom and bond economies, its cost- and time-effective balance, and its highly exploratory nature.

Classical multicomponent reactions are well known by their proper name and scope: Ugi [4,9], Passerini [6,10], van Leusen [11,12], Strecker [13], Orru [14], Mannich [6,15], Biginelli [6,16,17] and Hantzsch syntheses [6,18] need to be mentioned. However, the discovery of new multicomponent reactions is an active research field [19,20,21,22,23,24,25,26,27], with novel exciting MCRs being discovered and incorporated to the synthetic armamentarium every year. Among these MCRs with transition metal catalysis [28,29,30,31,32,33,34], radical process [7,21,35,36] and organo-catalysis [37,38,39,40,41] should also be mentioned.

Overall, MCRs can be subcategorized into two main classes, isocyanide-based multicomponent reactions (IMCRs) and non-isocyanide-based multicomponent reactions (NIMCRs).

**Figure 2:**
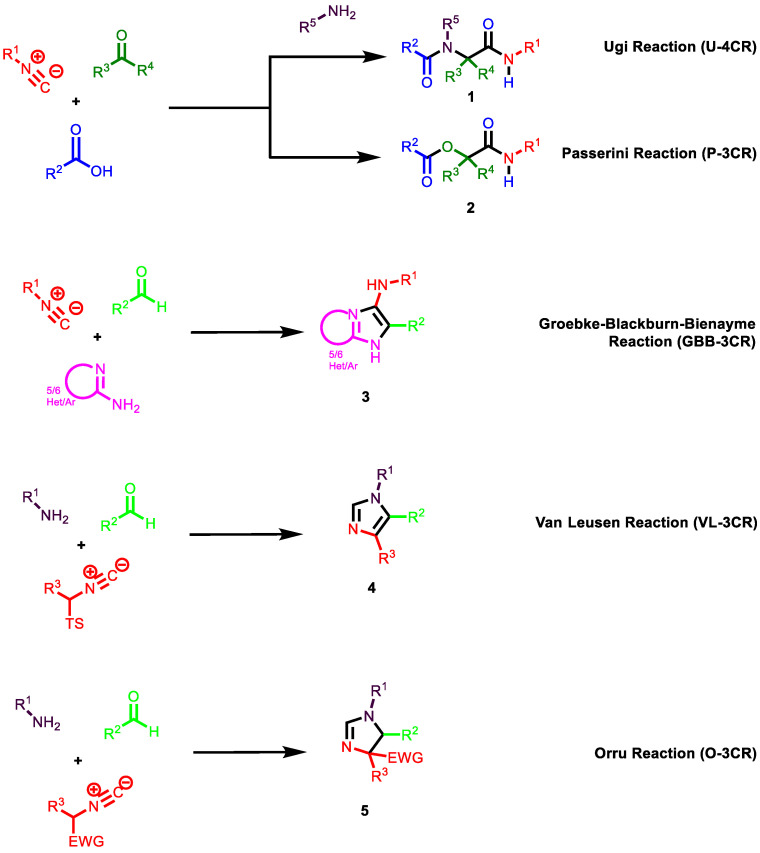
Isocyanide-based multicomponent reactions.

IMCRs are generally more versatile and often show high levels of regio- and chemo-selectivity, while the development of stereoselective IMCRs remains challenging. Isocyanides, the key component of IMCRs, have unique reactivity profiles as they can exhibit both nucleophilic and electrophilic behavior in organic reactions. Thus, isocyanides are a popular type of reactant for the progress of innovative MCRs in the rapid synthesis of valuable compounds [14,42,43,44,45,46]. Among them, the classical Passerini and Ugi reactions must be highlighted. The Passerini reaction (P-3CR) is a well-known IMCR involving a reaction between carboxylic acids and aldehydes or ketones and isocyanides to afford α-acyl carboxamides in one step [47,48] (Figure 2). The second most important IMCR is the Ugi reaction (U-3CR). This elegant four-component synthesis is a reaction between isocyanides, carboxylic acids, ketones or aldehydes and primary amines to afford dipeptide-like structures [49,50] (Figure 2).

**Scheme 1:**
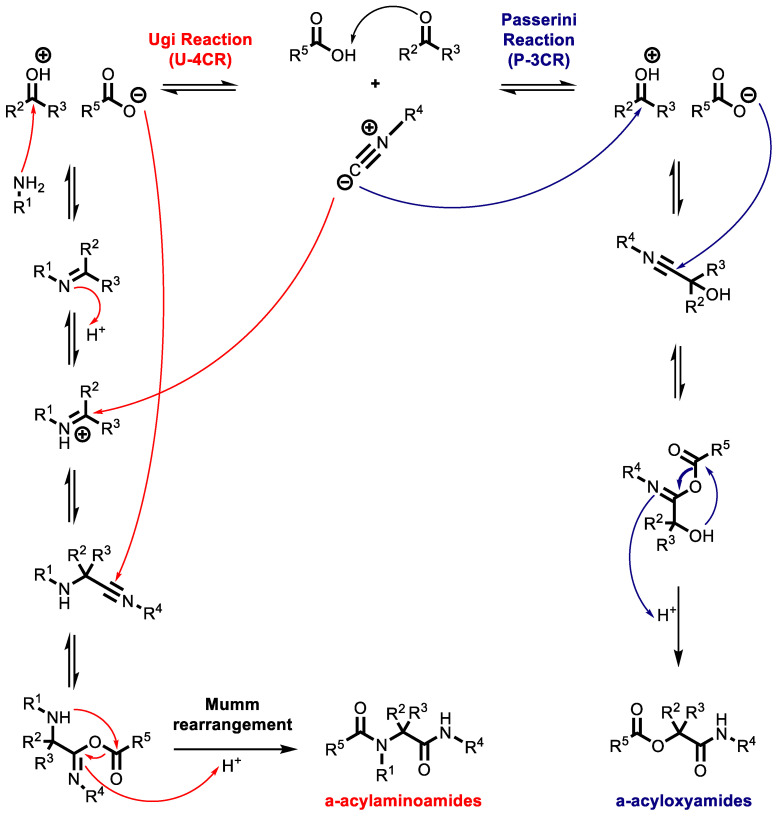
Mechanism of Ugi and Passerini reactions.

The mechanism of the Ugi reaction involves the reaction of a Schiff base reacting with a nucleophile and an isocyanide, followed by a Mumm rearrangement. In the related Passerini reaction, lacking the amine, the isocyanide reacts directly with the carbonyl group, but the other aspects of the reaction are the same. This reaction can take place concurrently with the Ugi reaction, acting as a source of impurities. These reaction mechanisms are shown in Scheme 1.

**Figure 3:**
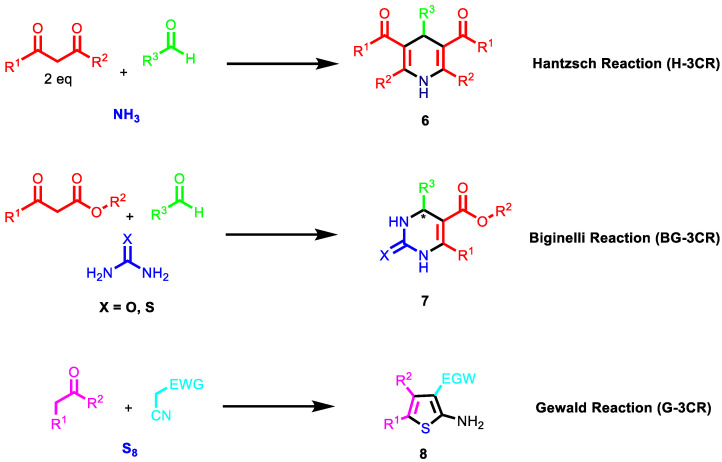
Non-isocyanide-based multicomponent reactions.

While both transformations are functional and mechanistically linked, the Ugi reaction is much more versatile than the Passerini reaction. The differences are not only based on the library size or feasibility, but mostly derive from the scaffold diversity. Both the Passerini and Ugi reactions lead to interesting peptidomimetic compounds that are potentially bioactive. Moreover, both reactions offer cost-effective and rapid access to generating large molecular libraries. The diversification of the starting compounds and the subsequent cyclization approaches provide a plethora of heterocyclic cores that have unequivocally contributed to the popularization of MCRs within the medicinal chemistry community. Other important IMCRs, depicted in Figure 3, are the Groebke–Blackburn–Bienayme reaction (GBB-3CR), Van Leusen reaction (VL-3CR), and Orru reaction (O-3CR). All of these reactions derive from further modifications of the Ugi reaction. The Groebke–Blackburn–Bienayme reaction involves the in situ formation of iminium species, followed by a non-concerted cycloaddition with an isocyanide to give the corresponding fused imidazoles. GBB-3CR is used for the one-pot synthesis of fused imidazole, bridgehead nitrogen heterocyclic compounds formaldehyde, isocyanide and amidine building blocks [51,52]. The Van Leusen-MCR (VL-3CR) is very useful for the synthesis of poly-substituted imidazoles in one pot from aryl substituted tosylmethylisocyanide (TosMIC) reagents and imines generated in situ [53]. Orru-3CR is the three-component condensation between an amine, an aldehyde and an α-acidic isocyanide, which efficiently provides highly substituted 2-imidazolines in a one-pot reaction [54,55,56].

Even though the classical IMCRs are not suited for generating heterocyclic compounds, new variations of the Passerini three-component reaction [48,57], Ugi three- and four-component reaction (U-4CR) [58,59], van Leusen reaction [60] and Groebke–Blackburn–Bienaymé reaction (GBBR) [61,62,63,64] have been explored and, indeed, IMCRs are leading today’s MCR chemistry [65]. For further information on isocyanide chemistry, the reader is directed toward further comprehensive review articles from Prof. Heravi’s group [66,67,68,69,70], Prof. Dömling’s group [71,72,73,74,75,76,77,78,79,80,81,82] and other extensive publications [83,84,85,86,87,88,89,90,91,92,93,94,95].

Despite its fascinating characteristics, there are several drawbacks to the use of isocyanides in organic synthesis: (1) it is harmful for the environment; (2) lability; and (3) suspected toxicity [96]. Due to these shortcomings, a practical solution was required for their wider use and further progress in isocyanide chemistry. Recently, research breakthroughs have been achieved in this direction under the title “in situ generated”, “isocyanide-less” or “odourless” isocyanide chemistry. It involves the formation of isocyanides using precursors and a subsequent reaction with other building blocks in the same pot to avoid the storage, separation and purification of isocyanides [97,98,99,100].

**Figure 4:**
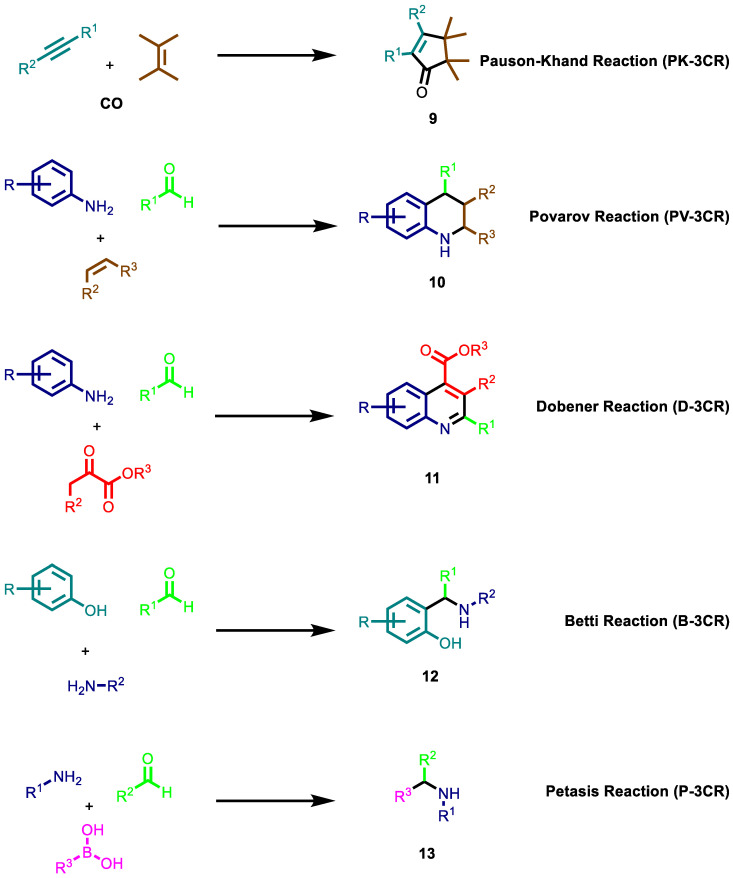
Other non-isocyanide-based multicomponent reactions reactions.

NIMCRs usually involve activated carbonyl compounds. An earlier example of such reactions is the Hantzsch reaction [101]. The reaction is performed with two equivalents of β-ketoesters/1,3 diketones, aldehydes and ammonia to afford symmetric dihydropyridines. Another non-isocyanide-based multicomponent reaction is the Biginelli reaction (BG-3CR) [102], which involves the synthesis of 3,4-dihydropyrimidin-2(1H)-ones from aldehydes, 1,3-dinucleophiles and β-ketoesters (Figure 4). In contrast to the previous reaction discussed, this reaction generates asymmetric products, obtaining at least two enantiomers per synthesis. In addition, depending on the 1,3-dinucleophile, more than one regioisomer can be generated. This feature allows the generation of large libraries of compounds in a short lap of time.

**Scheme 2:**
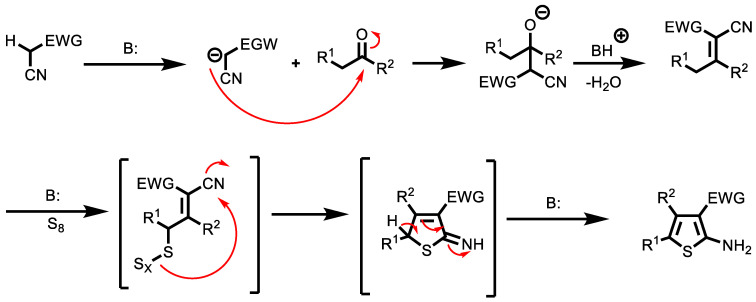
Mechanism of Gewald reaction.

Among NIMCRs, those involving cyanoacetic acid derivatives are extremely versatile, capable of affording chemically diverse scaffolds. NIMCRs usually involve Knoevenagel-type condensations of the cyanoacetic acid derivative with an aldehyde or ketone, followed by a Michael attack of a nucleophile and subsequent ring closure via a second nucleophile attack toward a nitrile. An example is the Gewald reaction (G-3CR) (Figure 4 and Scheme 2), which has recently gained ground with the usage of cyano acetamides. The G-3CR of cyanoacetic acid derivatives, active methylene carbonyls and elemental sulphur is a popular MCR often used in drug discovery yielding 2-amino-3-carbonyl thiophenes. Additionally, Gewald products can be easily transformed into more complex scaffolds by secondary transformations.

The Pauson–Khand reaction (PK-3CR) is also a versatile NIMCR, enabling the rapid assembly of α,β-cyclopentenones through [2 + 2 + 1] the cycloaddition of an alkyne, an alkene and carbon monoxide. For the generation of a diverse scaffold, other NIMCRs implemented are, namely, Pavarov-3CR, Dobener-3CR, Betti-3CR, and Petasis-3CR (Figure 4). For further information on NIMCRs chemistry, the reader is directed toward comprehensive review articles and other very interesting recent publications [89,90,92,93,103,104,105,106,107,108,109,110,111,112,113].

## 2. Evolution of Cancer Treatment

Cancer is a process of unregulated cell growth that determines the formation of a tumor that grows abnormally (usually for years), determining first a local disease that can metastasize, thus disabling other organs or vital functions. Cancer is considered a multifactorial disease due to the interplay between numerous determinant agents, such as genetic mutations, pollution, foods contaminants, viruses, chemicals and ionizing radiation [114].

Cancer is one of the leading causes of death worldwide [115] and accounts for 8–10 million deaths each year [116]. It has been estimated that, by 2025, there will be approximately 20 million new cancer patients worldwide per year [117].

**Figure 5:**
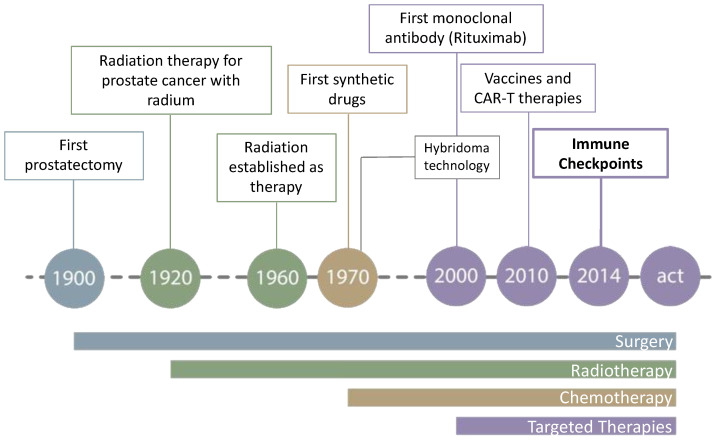
Evolution of cancer treatment.

Cancer treatment has been evolving since the beginning of the 20th century. The first and most obvious solution to treat a solid tumor was surgery; however, the therapy has evolved into less invasive, non-surgical treatments focusing on the destruction of tumors by radio and chemotherapy (Figure 5). 

Although relatively effective, they are not highly selective and might damage healthy tissues. As a result, targeted therapies have emerged, with new therapeutic targets of a very diverse nature [118]. This, coupled with the diversity of the known cancer types, makes the rapid and effective search for anticancer agents essential.

## 3. Anticancer Compounds Obtained from MCRs Approaches

In this chapter, representative and recent (from 2014) examples of the exploitation of MCRs for the discovery and optimization of antineoplastic drugs are selected. The selected case studies are classified according to the MCR employed in the publication.

### 3.1. Passerini Reaction

**Scheme 3:**
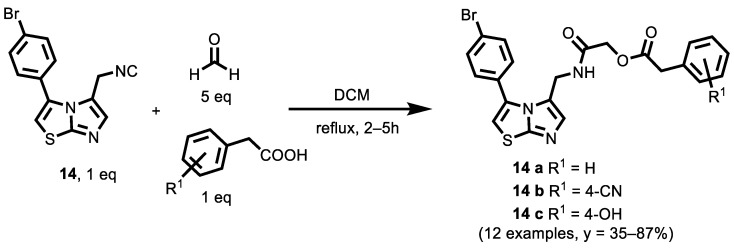
Three-Component Passerini Synthesis of α-acyloxyamides.

In 2021, Griglio et al. [119] synthesized a series of imidazothiazole derivatives based on a compound **14** (IC_50_ = 77 nM) previously synthesized by Tojo et al. [120], employing the Passerini multicomponent reactions (Scheme 3) and Ugi multicomponent reaction (illustrated in the next paragraph), and studied their inhibitory activities against human Indoleamine 2,3-dioxygenase 1 (rhIDO1). The ability of the selected compounds to inhibit rhIDO1 enzyme activity was determined in an in vitro cell-based assay in order to evaluate their inhibitory effect, together with their ability to permeate the cell membrane. The human melanoma A375 cell line was selected for the cellular assay because it does not express either the rhIDO1 gene or protein under normal culture conditions.

Acyloxyamides, in general, showed a better inhibitory activity compared to the corresponding a-acylaminoamides (illustrated in the next paragraph). Electron-withdrawing groups (R^1^), such as nitro and chlorine, gave rise, at any position, to a loss of activity. The cyano group or hydroxyl groups, when present at position 3, did not produce a remarkable effect on IDO1 inhibition, whereas, when displayed at position 4, they produced a significant increase in activity. Some of these compounds displayed IC_50_ values below 1.0 μM against the rhIDO1 enzyme; compounds **14 a**, **14 b** and **14 c** displayed enzymatic IC_50_ values of 0.58 μM, 0.24 μM and 0.20 μM, respectively. This work describes the multicomponent approach as a straightforward tool to rapidly access IDO1 inhibitors, providing a new direction for their future design and development.

**Scheme 4:**
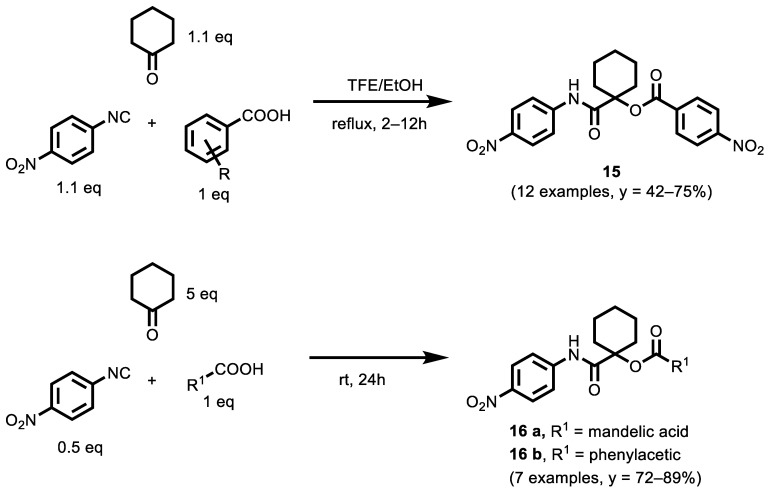
Three-Component Passerini Synthesis of α-acyloxycarboxamides.

In 2019, Ayoup et al. [121] reported a new series of Passerini products, which were synthesized and evaluated as potent caspase-dependent apoptotic inducers. A series of α-acyloxy carboxamides was prepared from p-nitrophenyl isocyanide, cyclohexanone and various carboxylic acids, and the principal amide-based scaffold was decorated by diverse substituents in good yield (Scheme 4). All of the synthesized compounds were screened for cytotoxicity against normal human fibroblasts and for their potential anticancer activities against three human cancer cell lines: MCF-7 (breast), NFS-60 (myeloid leukemia), and HepG-2 (liver) utilizing 3-(4,5-dimethylthiazol-2-yl)-2,5-diphenyl-2H-tetrazolium bromide (MTT) assay.

Among the tested compounds, many have IC_50_ values against different cell lines that are better than the reference compound. Some of them also have a good value of selectivity index (SI) and, therefore, of safety.

Compounds **15**, **16 a** and **16 b** were more potent than doxorubicin against the MCF-7 (IC_50_ = 0.0087 µM, 0.0050 µM and 0.0077 µM, respectively), NFS-60 (IC_50_ = 0.0077 µM, 0.0078 µM and 0.0081 µM, respectively) and HepG-2 (IC_50_ = 0.0141 µM, 0.0077 µM and 0.0141 µM, respectively) cancer cell lines; in addition, these compounds were the safest ones, showing the highest selectivity profiles in comparison with the other investigated compounds and doxorubicin against the NFS-60, MCF-7 and HepG-2 cell lines. The three compounds significantly induced apoptosis by caspase activation in all of the screened cancer cell lines utilizing flow cytometric analysis and a caspase 3/7 activation assay. Both compounds **15** and **16 a** were more potent caspase 3/7 activators than doxorubicin, while **16 b** showed comparable results. According to the physicochemical parameters, the in silico results showed that compounds **15**, **16 a** and **16 b** may be considered as drug-like candidates.

**Scheme 5:**
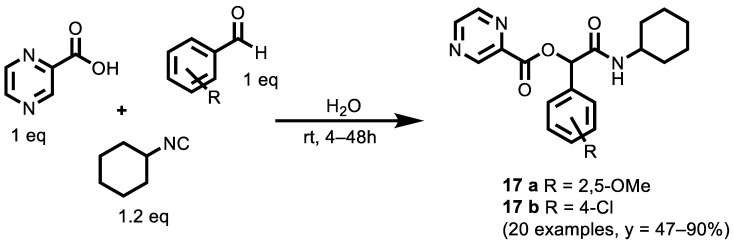
Three-Component Passerini Synthesis of α-acyloxyamides derived from pyra-zine-2-carboxylic acid (PA).

Pyrazine-2-carboxylic acid (PA), due to its heteroaromatic ring, can be explored as an anticancer agent. In a very recent paper [122], a series of twenty novels PA derivatives were synthesized using the Passerini reaction. Their cytotoxic activity was evaluated against three different cancer cell lines, including lung (A549), breast (MCF-7) and colon (HT-29); cisplatin was used as a reference drug. The general synthetic strategy employed to synthesize the PA derivatives was based on the Passerini multicomponent reaction using isocyanide, different aldehyde derivatives and PA (Scheme 5).

Almost all of the synthesized compounds showed higher anti-proliferative activity than PA, and resulted in a satisfying selectivity of the compounds between the cancerous cell and the non-cancerous cell line (MRC5). In all the studied cancer cell lines, the substitution of R with electron withdrawing groups such as halogens (Cl > Br > F) could affect the antitumor activity of compounds. When electron donor groups, such as methoxy (OCH3), was embedded as R, the resulting compound showed significant cytotoxic activity against the studied cancer cell lines. Among the twenty synthesized compounds, compounds **17 a** and **17 b** had high efficacy in suppressing cell growth and were considerably more potent in vitro cytotoxic activity than cisplatin and the others. **17 b** has shown promising anticancer activity, with IC_50_ of 6.11, 10.64 and 14.92 μM against the A549, MCF-7 and HT-29 cell lines, respectively. Moreover, **17 a** showed moderate cytotoxicity with IC_50_ of 14.09, 8.90 and 16.38 μM, against the A549, MCF7-7 and HT-29 cell lines, respectively (cisplatin IC_50_ was 9.37, 15.72 and 89.48 μM against the A549, MCF-7 and HT-29 lines, respectively). An electrophoretic mobility shift assay revealed that at higher concentrations, compound **17 b** was able to initiate reactive oxygen species (ROS)-induced DNA cleavage in the presence of H_2_O_2_ (1.0 mM). Furthermore, compounds **17 a** and **17 b** induced apoptosis in A549 cells, and these compounds can play an essential role in the future treatment of cancer.

**Scheme 6:**
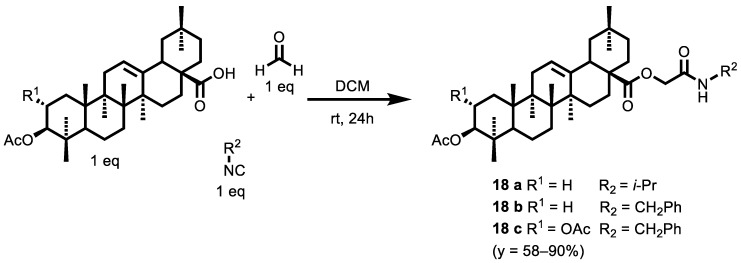
Passerini products from natural triterpenoids: oleanolic acid (OA) and maslinic acid (MA).

Natural products are often an inspiration for developing new drugs [123,124,125,126]. Moving toward this direction, Wiemann et al. [127] directed their attention to the combination of natural products and their derivatization by isocyanide-based multicomponent reactions (IMCRs). As a result, a set of 3–4 CR Ugi (illustrated in the next paragraph) and 3CR Passerini products (Scheme 6) were successfully synthesized from natural triterpenoids, i.e., oleanolic acid (OA) and maslinic acid (MA), followed by a biological evaluation of the novel α-acylamino carboxamides and the α-acyloxy carboxamides in colorimetric SRB assays to determine their cytotoxicity.

In comparison to U-4CR, an increased reaction rate was observed, likely due to the increased solubility of the acid component in dichloromethane (DCM). The biological activity of these compounds was examined in colorimetric sulforhodamine B (SRB) assays, and the EC_50_ values were determined for six human tumor cell lines: 518A2 (melanoma), A2780 (ovarian carcinoma), HT29 (colorectal carcinoma), MCF7 (breast carcinoma), A549 (lung adenocarcinoma), 8505C (thyroid carcinoma) and nonmalignant mouse fibroblasts (NIH 3T3). The EC_50_ values were comparable to those measured for both the Ac-OA and Ac-MA Ugi examined. Compounds **18 a**, **18 b** and **18 c** showed a range of EC_50_ = 8.8–20.0 µM, EC_50_ = 6,4–11.3 µM and EC_50_ = 1.0–7.3 µM, respectively. However, a marked decline in the selectivity index was observed, thus reducing the pharmacological value of these α-acyloxy carboxamides.

**Scheme 7:**
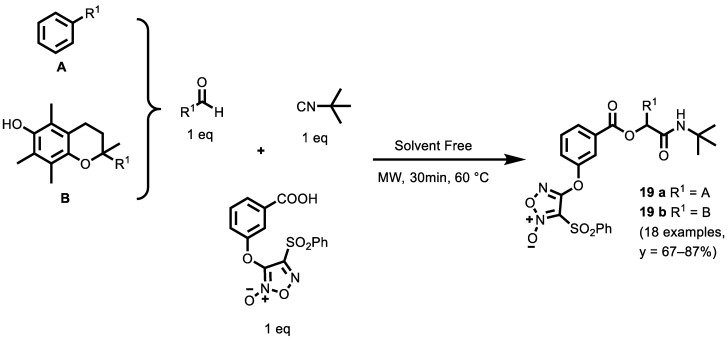
Three-Component Passerini Synthesis of NO-Donor Compounds.

In 2019, Ingold et al. [128] focused their attention on a library of NO-donor compounds through the Ugi Reaction (illustrated in the next paragraph) and the Passerini reaction with optimized yields under green chemistry conditions (Scheme 7). Nitroxyl or furoxanyl groups were incorporated into the carboxylic acid component to evaluate whether the position of the NO-donor moiety plays a fundamental role in the biological activity compared to the previous series and, thus, to explore the chemical space in search of new chemotypes with improved antitumor activity. The use of green solvents, such as water or ethanol, either at room temperature or under microwave irradiation (MW) was productive and it was also observed that the reaction could be performed under solvent-free conditions, using MW irradiation, ultrasound or conventional heating. The authors have selected as our standardized conditions: neat with MW irradiation in a closed vessel for 30 min at 60 °C.

Compounds have also been, in some cases, Boc-deprotected to increase the functional diversity of the library for structure–activity relationship (SAR) studies. Passerini derivatives **19 a** and **19 b**, each containing a furoxan system as a NO-donor, are potent anticancer agents, exhibiting GI_50_ values against all cells in the range 0.021–5.8 μM. Compound **19 a** was almost seven times more active than cisplatin and etoposide against breast cancer cells HBL-100, and almost 30 times more potent than cisplatin and almost 140 times more potent than etoposide against SW1573 cancer cells. This compound, furthermore, exhibited lower potency against noncancerous human keratinocytes (HaCaT), indicating a selectivity against cancerous cells. In addition, compound **19 a** was able to release NO in HeLa cells, and the antiproliferative activities declined with the increasing concentration of scavenger (hemoglobin), suggesting a potential role of NO release in their anticancer mechanism. Future studies should obtain a deeper insight into their antiproliferative mechanism, evaluate the antiproliferative activity of isolated isomers and determine their in vivo activity.

### 3.2. Ugi Reaction and Its Modifications

**Scheme 8:**
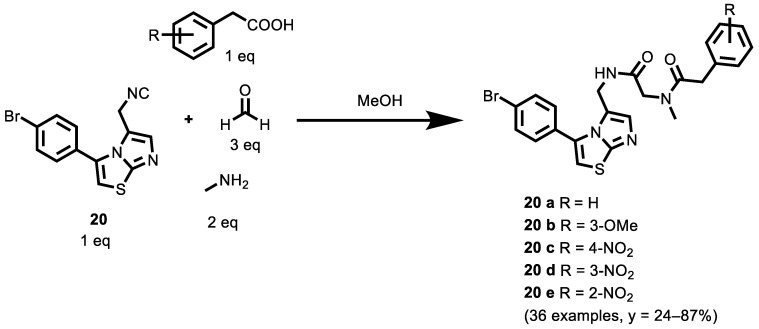
Four-Component Ugi Synthesis of α-acylaminoamides.

In parallel with the modifications of compound **20** (Scheme 8) through the Passerini reaction in order to obtain α-acyloxyamides derivatives (displayed above), Griglio et al. [119] synthesized α-acylaminoamides through the Ugi reaction. In addition, in this case, the compounds were tested for inhibitory activities against rhIDO1. In general, this type of derivative shows a lower inhibitory activity than the α-acyloxyamides derivatives, even if they express values in the micromolar range. In fact, compounds **20 a**–**20 e**, shown in Scheme 8, displayed enzymatic IC_50_ value of 0.69 μM 0.45 μM, 0.81 μM, 0.63 μM and 0.73 μM, respectively (see compounds 14 a and 14 b in the previous paragraph: 0.24 μM and 0.20 μM). The effect of different substituents on the benzyl ring was investigated.

The cyano group produced a complete loss of activity when introduced at positions 3 and 4. The same occurred when the hydroxyl moiety was introduced at position 2; on the contrary, the hydroxyl group at position 3 gave rise to the most active compound of this series (**18 b**), with an IC_50_ value of 0.45 µM. The presence of a nitro group produced a progressive increase in the activity by moving the substituent from position 4 (**20 c**, IC_50_ = 0.81 µM) to either position 2 (**20 e**, IC_50_ = 0.73 µM) or 3 (**20 d**, IC_50_ = 0.63 µM). The presence of a chlorine at different positions on the benzylic ring did not produce any improvement in the activity.

**Scheme 9:**
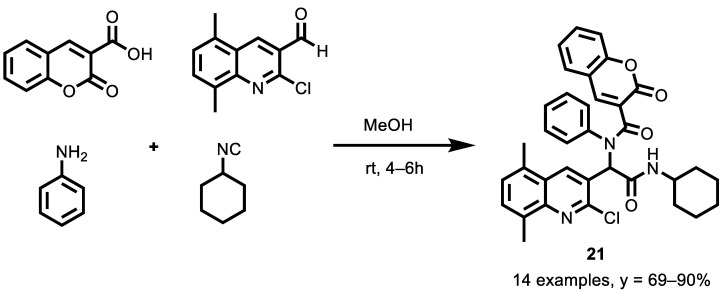
Formation of diamide Ugi reaction compounds.

An efficient four-component Ugi synthesis of quinoline-coumarin hybrids was described by Taheri et al. [129]. This simple access to quinoline-coumarin derivatives implicated the use of coumarin-3-carboxylic acid, diverse 2-chloroquinoline-3-carbaldehydes, aniline derivatives and aliphatic isocyanides in methanol at room temperature (Scheme 9).

The cytotoxic effects of fourteen products were investigated in A2780 human ovarian cancer cells. Several methods were also performed, including cell viability, the induction of apoptosis, a mitochondrial membrane potential (MMP) assay, the determination of intracellular ROS, caspase 9 and a 3 activity assay. Interestingly, compound **21** showed excellent anticancer activity, with an IC_50_ value of 25 µg/mL (0.042 µM). A2780 cells were treated with various concentrations of hybrids (0–100 μg/mL) for 24 h and the cell viability was evaluated by a 3-(4,5-dimethylthiazol-2-yl)-2,5-diphenyl-2H-tetrazolium bromide (MTT) growth inhibition assay; doxorubicin was chosen as a positive control (IC_50_ = 6.1 µM). Furthermore, the synergistic effect of compound **21** on apoptosis induction might be due to the regulation of anti-apoptotic agents, including the down-regulation of Bcl-2 and survivin and the activation of caspase 9 and 3. Further examinations indicated that compound **21** increased the ROS levels, reduced the MMP, and induced apoptosis in the A2780 cells through the intrinsic mitochondrial pathway.

**Scheme 10:**
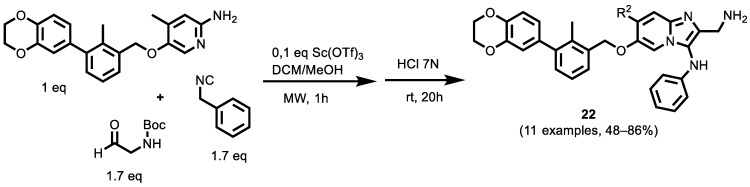
Groebke–Blackburn–Bienaymé products of imidazo[1,2-a] pyridine-based inhibitors.

In 2021, Butera et al. [130] focused their attention on novel therapeutic modalities, such as small molecules or peptides, through MCR synthesis. A small library of compounds was synthesized using the GBB-3CR, resulting in the structure–activity relationship of imidazo [1,2-a]pyridine-based inhibitors (Scheme 10). These inhibitors were tested for their biological activity using various biophysical assays, giving potent candidates with low-micromolar PD-L1 affinities.

The authors found the optimal reaction conditions for the used substrates in the presence of scandium triflate (10 mol%) as a catalyst, 2:1 DCM/MeOH as the solvent system, a concentration of 0.3 M concerning to the amidine, and 1.7 equivalent of the isocyanide and aldehyde components. Microwave-assisted heating for 1 h generated the corresponding GBB products in good to excellent yields (48–86%) The products were tested for their antiproliferative activity through various biological assays, exhibiting IC_50_ values of 16.8–1.8 μM (compound **22**) at a concentration of 50 μM. These results open the door to an interesting bioactive scaffold that could lead to a new class of PD-L1 antagonists.

**Scheme 11:**
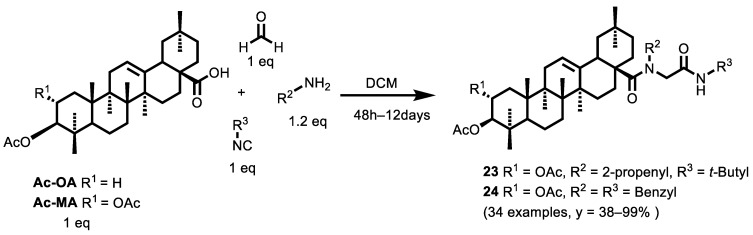
Ugi products from natural triterpenoids: oleanolic acid (OA) and maslinic acid (MA).

Wiemann and Csuk [127], in addition to the synthesis of the α-acyloxy carboxamides (illustrated in the paragraph above), also synthesized a library of 34 α-acylamino carboxamides from natural triterpene through U-4CR (Scheme 11).

First, they studied the U-4CR derivation of oleanolic acid: the isocyanide used was a *tert*-butyl isocyanide and the amino component (R^2^) was varied (methyl, 2-propenyl, 3-furanyl-methyl, butyl, isopropyl, benzyl, 3-fluorobenzyl). Furthermore, different isocyanide groups were also used, such as *n*-butyl isocyanide or *i*-propyl isocyanide or benzyl isocyanide and methyl isocyanoacetate (while the amine component remained unchanged for better comparability of the biological results).

Due to the low solubility of triterpenoic acids in polar-aprotic solvents, the reactions were characterized by long reaction times and moderate conversions. However, the increase in the reaction temperature and the use of MW did not result in higher efficiencies, but the reaction time could be reduced to 2 days from 7–12 days. Secondly, they studied the U-4CR derivation of maslinic acid. Due to their better solubility, the derivatives were synthesized in one step directly from 2,3-di-O-acetyl-MA (Ac-MA). In addition, in this case, the biological activity of these compounds was investigated in SRB colorimetric assays, and the EC_50_ values were determined for six human tumor cell lines: 518A2 (melanoma), A2780 (ovarian carcinoma), HT29 (colorectal carcinoma), MCF7 (breast carcinoma), A549 (lung adenocarcinoma), FaDu (hypopharyngeal carcinoma with multidrug resistance) and nonmalignant mouse fibroblasts (NIH 3T3). All of the compounds showed moderate to good cytotoxicity for several human tumor cell lines. In particular, compounds **23** (R^1^ = OAc, R^2^ = 2-propenyl, R^3^ = *t*-butyl) and **24** (R^1^ = OAc, R^2^ = R^3^ = benzyl) gave remarkable EC_50_ values in the low μM range (0.3 µM and 0.4 µM, respectively, for ovarian carcinoma cells A2780) and high tumor/non-tumor cell selectivity. Further biological assays showed that treating A2780 cells with compounds **23** or **24** led to a non-necrotic, controlled cell death, in part occurring through the pathway of apoptosis. In addition, Wiemann and Csuk also highlighted how varying the substrate yielded acetylated products and compounds holding a terminal benzylamide moiety with higher selectivity, while the presence of ester groups initiated a loss of this selectivity.

**Scheme 12:**
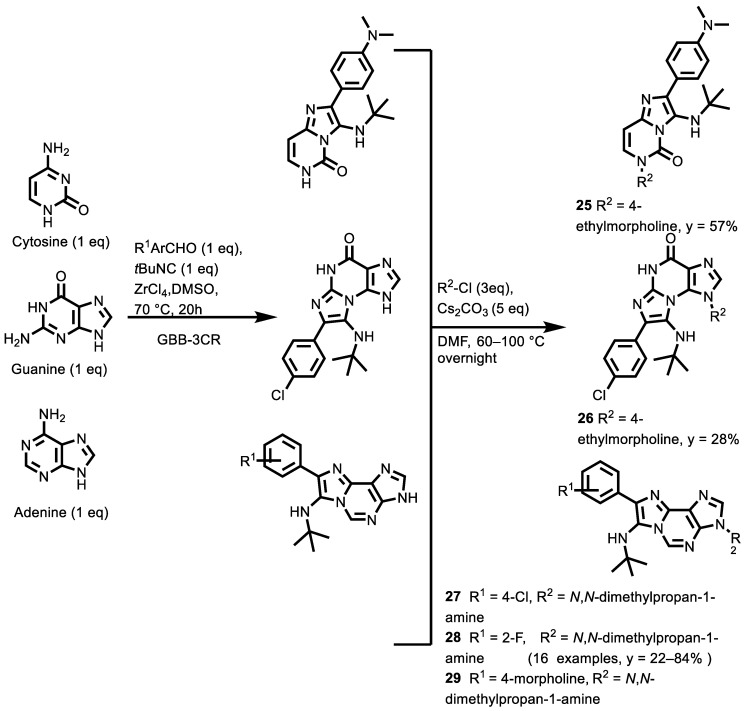
Groebke-Blackburn-Bienaymé-mediated three-component synthesis of imidazo[2,1-i]purine derivatives.

In 2020, for the investigation of new drug-type G-quadruplex selective binders, Pelliccia et al. [131] used the design of nucleobases as a starting point as synthons in a multicomponent reaction. Thus, a series of multifunctional imidazo[2,1-i]purine derivatives were synthesized via a convergent GBB-3CR of amino-aza-heterocycles, benzaldehydes and isocyanide, followed by an SN_2_ with various aminoalkyl chlorides [R^2^ = 4-morpholinyl(CH_2_)_2_, pyrrolidinyl(CH_2_)_2_, Me_2_N(CH_2_)_3_, *N*-phthalamidyl(CH_2_)_2_] (Scheme 12).

The compounds **25**, **26** and **27**–**29** were endowed with a small molecular weight (≅500 Da), water solubility, easy functionalization, and a selectivity profile as G-quadruplex binders as safe and effective anti-cancer agents, as well as being non-toxic toward non-cancerous cells. Compounds, **27**, **28** and **29**, were selected as the best performing ones, both in terms of their potency and selectivity toward BCL2 and c-MYC G4s, over the other G4 topologies screened and a duplex model. Indeed, compound **27** exhibited selective antiproliferative action (IC_50_ = 17 µM) toward Jurkat human T lymphoblastoid cells, without showing significant cytotoxicity toward normal cells, such as skin-derived cells. Furthermore, a downregulation at both the mRNA and protein levels was observed through qPCR and Western blot analyses, in which the cells were treated with the selected compounds. On the basis of both the herein-reported biophysical and biological data and the poor availability in the literature of selective BCL2 G4 ligands, compound **27** represents a promising advancement toward the pathway of the specific targeting of cancer cell lines by means of gene promoters’ G4 binders.

**Scheme 13:**
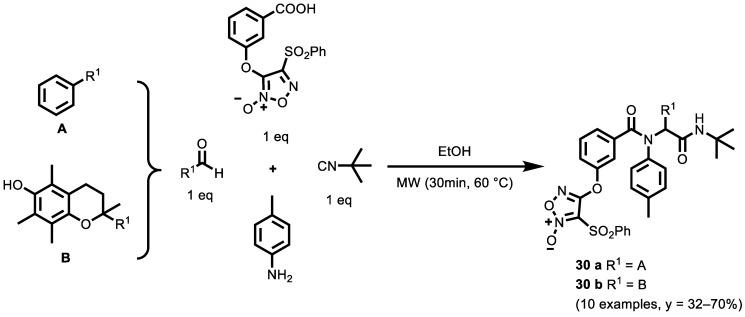
Ugi reaction for synthesis of NO-Donor Compounds.

Moreover, Ingold et al. [128] also obtained α-acylaminoamides derivatives (compare α-acyloxyamides derivatives in the paragraph above) under green conditions through the Ugi reaction (Scheme 13).

The optimized experimental conditions for the Passerini reaction (see respective paragraph in chapter 3.1 Passerini Reaction) failed to deliver the Ugi adduct. For this reason, the IMCRs were optimized and performed (under eco-friendly conditions) in EtOH and MW irradiation (closed vessel, 30 min, 60 °C). The compounds were also, in some cases, Boc-deprotected to increase the functional diversity of the library for SAR studies. The biological activity of these compounds and their cytotoxicity against six human solid tumor cell lines (A549 (lung), HBL-100 (breast), HeLa (cervix), SW1573 (lung), T-47D (breast), WiDr (colon) have been studied. Compounds **30 a** and **30 b** exhibited notable activities against most of the tested cell lines, as revealed in their GI_50_ values being between 0.24–5.8 μM and 0.021–3.0 μM, respectively, against all of the tested cell lines, as well as their sensitivity against HBL-100 and SW1573 cell lines. Compound **30 a** was almost eight times more potent than cisplatin and etoposide against the resistant cell lines, T-47D and WiDr; moreover, it was extremely potent against alveolar cell carcinoma SW1573, being 140 times more active than cisplatin and 700 times more potent than etoposide.

Furthermore, compounds **30 a** and **30 b** were evaluated for their ability to release NO in cancer cells as a first approximation to their mechanism of action. Compound **30 a** was able to release NO in the presence of HeLa cells.

**Scheme 14:**
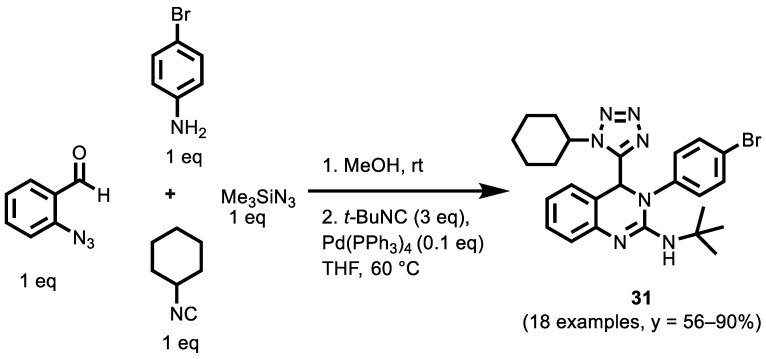
Ugi-4CR synthesis of dihydroquinazoline derivatives.

In a very recent publication, Xiong et al. [132] reported the synthesis of 4-tetrazolyl-3,4-dihydroquinazoline derivatives through a sequential Palladium-catalyzed azide-isocyanide cross-coupling/cyclization Ugi reaction, with good yields. The Ugi-azide reactions of various 2-azidobenzaldehydes (R^1^ = 4-F, 4-Cl, 4-CH_3_O and 5-Me), amines (R^2^ = Ph, 4-Cl-Ph, 4-Br-Ph, 4-CH_3_-Ph, 4-CH_3_O-Ph, 2-Cl-Ph, 2,4-dimethyl-Ph, *n*-Bu, *i*-Pr, *t*-Bu), trimethylsilyl azide and isocyanides (R^3^ = *t*-Bu, *c*-C_6_H_11_, 1-adamantyl, *n*-Bu) produced azide intermediates that were treated with diverse isocyanides (R^4^ = *t*-Bu, *c*-C_6_H_11_, 1-adamantyl, 4-CH_3_O-Ph, 4-Cl-Ph, MeO_2_CCH_2_, TsCH_2_) to afford compound 18 examples of dihydroquinazoline derivatives (Scheme 14).

With the advantages of mild reaction conditions, good yields and the easy availability of raw materials, this strategy shows the synthetic potential of combining isocyanide-based multicomponent reactions with azide-isocyanide coupling reactions to produce structural diversity and complexity. Among the synthesized compounds, compound **31** (R^1^ = H, R^2^ = 4-Br-Ph, R^3^ = *c-*C_6_H_11_, R^4^ = *t*-Bu,) was a promising starting point for the development of a breast cancer drug. The proliferation of breast cancer cells treated with **31** at the concentration of 0.3 µM was detected using the 5-ethynyl-2′-deoxyuridine (EdU) method. The results showed that, with the increase in **31** concentrations, the number of EdU positive cells in breast cancer decreased gradually. This evidence shows that compound **31** could significantly inhibit the proliferation, and promote the apoptosis, of breast cancer cells.

**Scheme 15:**
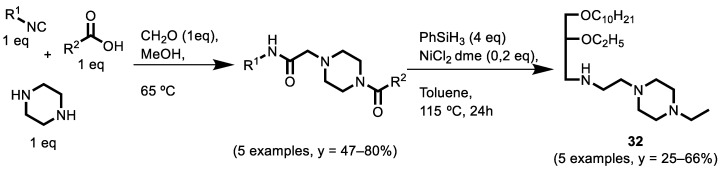
Synthesis of LPAs using *N*-split Ugi reaction.

In 2022, Nichugovskiy et al. [133] demonstrated for the first time that the anticancer activity of lipophilic synthetic polyamines (LPAs) with fragments of piperazine is comparable to that of aliphatic LPAs. In their publication, they demonstrated a new method for the synthesis of LPAs using the *N*-split Ugi multicomponent reaction and subsequent reduction in amide groups by PhSiH_3_ (Scheme 15). The anticancer activity was evaluated in the A-549, MCF7 and HCT116 cancer cell lines.

With the application of this method, the authors reduced the synthetic steps and increased the total yield of the LPAs from 7% to 28%. The use of PhSiH_3_ and NiCl_2_ (ethylene glycol dimethyl ether complex) effectively permitted us to reduce several amide groups in the PA precursors and has proven to be a very reliable and efficient method. The obtained LPAs manifest excellent preliminary biological activity in cancer cell lines, and among these, compound **32** showed the highest anticancer activity within all the tested cell lines: IC_50_ = 3.0 ± 0.4 µM (A-549 cell line), 1.0 ± 0.1 µM (MCF7 cell line) and 3.8 ± 0.5 µM (HCT116 cell line). These IC_50_ values are several times higher than that of cisplatin, which is used in medical practice.

### 3.3. Biginelli Reaction

**Scheme 16:**
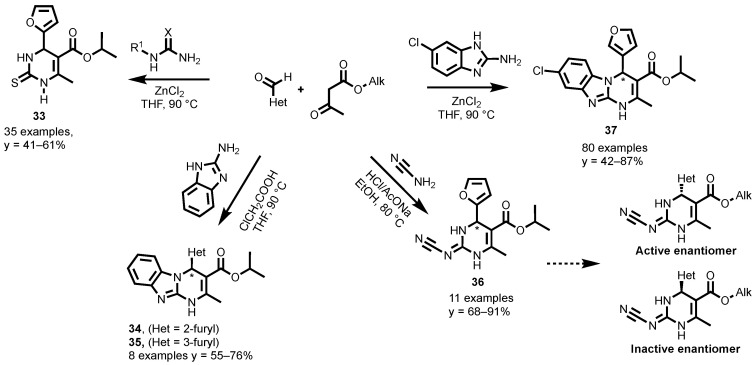
Three-Component Biginelli Synthesis of A_2B_ antagonists.

With the initiative to develop novel cancer immunotherapeutic agents via the modulation of the A_2B_ adenosine receptor, Sotelo et al. [134,135,136,137,138] discovered several families of (monocyclic, bicyclic or tricyclic) pyrimidine derivatives (Scheme 16) that were readily and efficiently assembled through a three-component Biginelli reaction. 

The synthesized compounds were evaluated for their antagonistic activity toward the Adenosine Receptors. Many compounds have yielded good K*_i_* values in A_2B_ (K*_i_* ≤ 25 nM) and excellent receptorial subtype selectivity profiles. The most promising for A_2B_ are the monocyclic compounds **33** with K*_i_* = 10.2 nM, **36** with K*_i_* = 24.3 nM; and tricyclic compounds **34** with K*_i_* = 3.49 nM; **35** with K*_i_* = 11.40 nM. During the study, some A_2A_/A_2B_ duals emerged, being of particular interest in cancer immunotherapy by acting synergistically on the immune system and tumor cells. The most promising is **37**, with K*_i_* = 176.2 and 6.1 nM for A_2A_ and A_2B_, respectively. The differences in the affinity of both enantiomers were evaluated first on **36**, and confirmed in subsequent works, showing that the affinity for A_2B_ (and A_2A_) is exclusively due to one of the two enantiomers (Scheme 16).

The administration of these compounds to breast cancer patient derived cells, was first revealed through a reduced tumor spheroids growth rate [137]. The study also demonstrated a marked effect on immune system cells, recovering from adenosine-mediated immunosuppression and increasing the production of proinflammatory cytokines. This effect in the immune system has been recently verified [138].

**Scheme 17:**
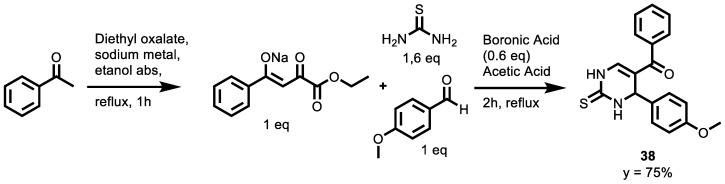
Synthesis of compound 38 of new dihydropyrimidines.

In 2021, a new ecofriendly Biginelli reaction procedure was adapted to prepare new dihydropyrimidines using β-aroylpyruvates as synthons [139]. All of the synthesized compounds were evaluated for their anticancer activity against 59 human cancer cell lines, representing different cancer types, including leukemia and melanoma, as well as lung, colon, ovarian, renal, prostate and breast cancer. The anticancer activity was demonstrated as a percentage growth inhibition of different human cell lines exerted by a single dose of 10^−5^ M. Compound **38** (Scheme 17) showed marked wide spectrum anticancer activity towards most of the tested cancer cell lines, with a percentage of growth inhibition of 29.04–71.68% upon a single dose administration (10^−5^ M). Specifically, most of the leukemia and colon cancer cell lines were highly sensitive to the antitumor effect of compound **38**, with a growth inhibiting effect exceeding 50%, as well as the colon cancer HT29 cell line (53.16%) and the leukemia cell lines, K-562 (64.97%) and SR (71.68%).

**Scheme 18:**
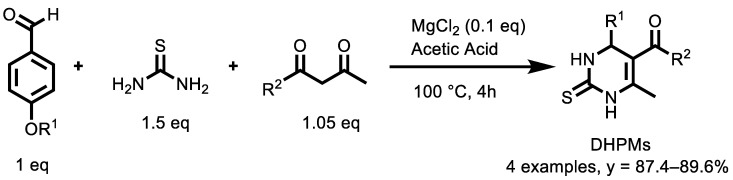
Three-component synthesis of dihydropyrimidinones group.

Recently, Li et al. [140] published on an interesting study ACS Macro Letters about the development of a polymer-drug strategy to explore anticancer polymers. These consist in a series of monomers containing groups with potential anti-cancer activity that were easily prepared by the Biginelli reaction. These monomers were used to produce water-soluble polymers by practical radical co-polymerization. A series of dihydropyrimidinones groups (DHPMs), similar to Monastrol, a promising anticancer agent which inhibits Eg5 kinesin protein that is highly expressed in many tumor cells and is closely related to the occurrence and development of tumors, was prepared through the Biginelli reaction (Scheme 18).

The obtained polymers are biocompatible and can be used directly to suppress the proliferation of various cancerous cells without the release of small molecules. In such experiments, one of the DHPMs, P4, a polymer with the best anticancer ability, effectively suppressed the proliferation of different cancer cells by inhibiting cell mitosis, similarly to Monastrol, confirming the rationale behind the theoretical calculations. The inhibition effect of P4 was investigated on different cells: a model of normal cells (L929), hepatoma cell line (SMMC-7721), human cervical cancer cell line (HeLa) and a human breast cancer cell line (MCF-7). The results suggested that P4 selectively inhibits the proliferation of cancer cells, rather than killing them. Furthermore, immunostaining was carried out to detect the inhibitory process of P4 during cellular proliferation, suggesting that P4 shares a similar anticancer mechanism of the small molecular monastrol and that it could be considered a potential anticancer polymer.

**Scheme 19:**
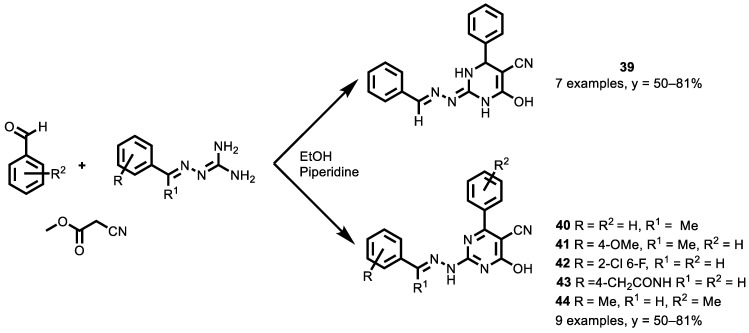
Three-component synthesis of pyrimidine derivatives.

In 2020, the study by Yavuz et al. [141] aimed at developing an effective, improved and easy method to synthesize substituted pyrimidine-5-carbonitriles, without the isolation of their intermediates. In a single reaction step, pyrimidine derivatives were synthesized from the triple reaction of aromatic aldehydes, ethyl cyanoacetate and a few guanyl hydrazone derivatives (Scheme 19). In this study, the reaction conditions were optimized, monitoring the order of addition of reagents and the best base to be used. The reaction conditions were first optimized using different bases, such as NaOH, K_2_CO_3_, NaHCO_3_ and piperidine. When NaOH (10% mol) was added into the reaction system, the products were obtained in moderate yields in both refluxing in ethanol and at room temperature, while when piperidine was added as a catalyst to the reaction medium, higher yield compounds were obtained with the same reaction conditions. First, aromatic aldehyde, ethyl cyanoacetate and a catalytic amount of piperidine were added to obtain the best yield.

The synthesized compounds were tested in vitro against the human colon cancerous cell line (DLD-1) and a human breast cancerous cell line (MDA-MB-231). Nearly all of those compounds showed cytotoxic activity in the tested cell lines. However, in general, the synthesized pyrimidine-based compounds exhibited better cytotoxicity against the colon cancer cell line than the tested breast cancer cell line. In particular, compounds **39**, **40** and **41** have a significant effect against DLD-1. Furthermore, compounds **42**, **43** and **44** exhibited lower IC_50_ values compared to the other synthesized compounds and were tested against MDA-MB-231.

On the other hand, in 2022, Ashma et al. [142] focused their attention on the synthesis of nicotinic acid hydrazide metal complexes and their potential anticancer and antibacterial activity, together with their ability to act as a catalyst. This work addressed newer bidentate Schiff bases, such as L5 and L6, and their metal complexes Co(II), Ni(II), Cu(II) and Zn(II) were synthesized by a one-pot method. The multicomponent reaction of Biginelli has been tested with a catalytic amount of their transition metal complexes, Co(II), Ni(II), Cu(II) and Zn(II), in the presence of a solvent. Hydrazone-substituted transition metal complexes Cu (II) showed excellent catalytic activity compared to the other derivatives. The metal complexes exhibited good anti-cancer activity, overall, in 400–800 μg for most L5 and L6 complexes, but Co, Cu and Zn showed higher activity at the nano gram scale for 24h treatment on the human colon cancer cell line HT29. The Zn(II) complex, in particular, exhibited greater efficacy, even at the 600 ng concentration, which makes it a potent candidate for further anti-cancer studies in different type of cancers cells.

### 3.4. Other MCRs for the Synthesis of Anticancer Drugs

**Scheme 20:**
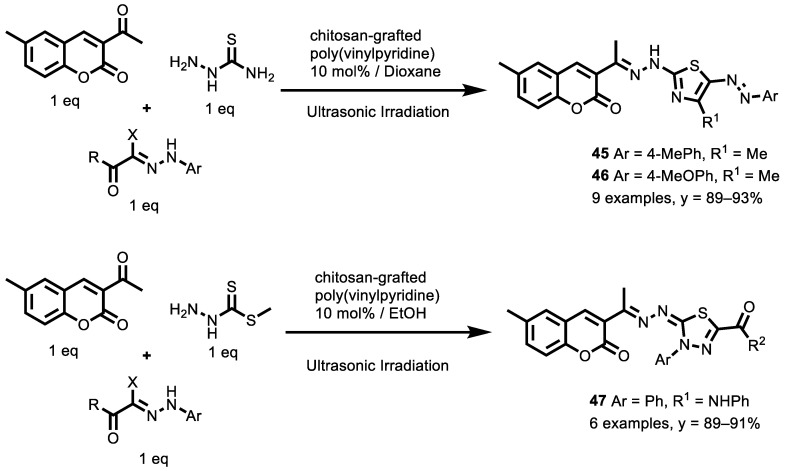
Three-component synthesis of thiazoles and thiadiazoles.

In a recent paper, Alshabanah et al. [143] reported the design and synthesis of thiazoles and thiadiazoles linked to position 3 of coumarin as novel 3-azolylcoumarins as potential anticancer agents, utilizing the sonication technique and using chitosan-grafted poly(vinylpyridine) as an eco-friendly catalyst (Scheme 20).

In this study, the best reaction conditions were evaluated. Regarding the base chitosan-grafted poly(vinylpyridine), 10 mol% was the best choice of a basic catalyst (respect to trietilammina and chitosan) under ultrasonic irradiation (USI). In addition, heating at 50 °C under USI was more efficient than conventional heating as it reduced the reaction time and increased the product yields for the thiazoles derivatives, but not for the thiadiazole (respect to the 25 °C). The cytotoxic activity of the newly prepared compounds was determined against the liver carcinoma cell line (HEPG2-1) using the MTT assay, and doxorubicin was used as a reference compound. Compounds **45** (Ar = 4-MePh, R^1^ = Me), **46** (Ar = 4-MeOPh, R^2^ = Me) and **47** (Ar = Ph, R^2^ = NHPh), with IC_50_ values of 0.43 µM, 0.29 µM and 0.48 µM, respectively, (doxorubicin IC_50_ = 0.31 µM) have promising cytotoxic activities. The 1,3-thiazole ring introduction of an electron-donating group (methyl or methoxy groups) enhanced the antitumor activity. In contrast, the introduction of the electron-withdrawing group (chlorine or bromine or nitro group) at C4 of the phenyl group at position 4 in the 1,3-thiazole ring decreased the activity. In addition, for 1,3,4-thiadiazoles, generally, on fixing the substituents at position 5, the electron-donating group (methyl) at C4 of the phenyl ring enhances the antitumor activity, while the electron-withdrawing group (chlorine) decreases the activity.

**Scheme 21:**
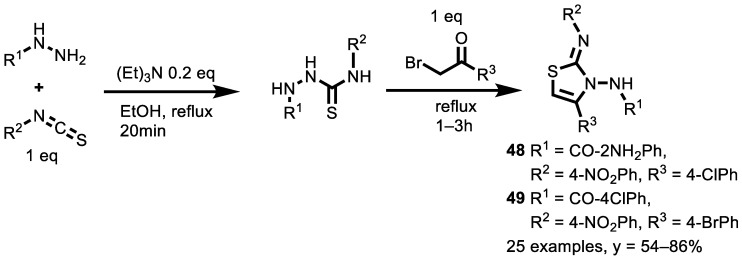
Three-component synthesis of aryl thiazoles derivatives.

In 2017, a time-efficient three-component synthesis of a series of substituted aryl thiazoles derivatives was achieved through the reaction between different (ortho, para) substituted benzoyl hydrazides and (ortho, meta, para) aromatic isothiocyanate, followed by aromatic α-halo ketone in the presence of triethylamine under reflux in ethanol (Scheme 21) [144].

In this study, twenty-five substituted aryl thiazoles were synthesized, and their in vitro cytotoxicity was evaluated against MCF-7 (ER+ve breast), MDA-MB-231 (ER−ve breast), HCT116 (colorectal) and HeLa (cervical). The activity was compared with the standard anticancer drug doxorubicin (IC_50_ = 1.56 ± 0.05 μM). Many of the twenty-five synthesized compounds were found to be cytotoxic for all four cancer cell lines, with IC_50_ values ranging between 5.37 and 46.72 μM. Compound **48** was highly toxic against the breast cancer cells, with IC_50_ = 5.37 ± 0.56 μM for MCF-7 (ER+ve breast), IC_50_ = 17.77 ± 4.32 μM for MCF-7 (ER+ve breast) and against the HCT116 (colorectal) cell line, with IC_50_ = 16.99 ± 0.94 μM. Nevertheless, compound **49** showed IC_50_ values of 10.96 ± 0.33 μM, 9.9 ± 0.59 μM and 8.28 ± 0.21 μM for MCF-7 (ER+ve breast), MDA-MB-231 (ER-ve breast) and HeLa (cervical), respectively. These compounds deserve to be further investigated in vivo as anticancer lead compounds.

**Scheme 22:**
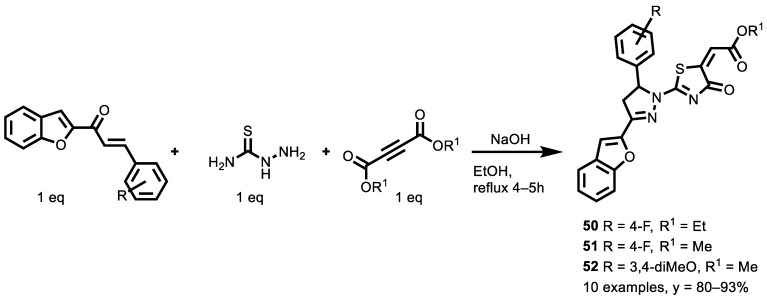
Three-component synthesis of pyrazolo-oxothiazolidine derivatives.

In 2018, Yakaiah et al. [145] obtained novel pyrazolo-oxothiazolidine derivatives through the condensation of 1-(benzofuran-2-yl)-3-(substituted-arylprop-2-en-1-ones), thiosemicarbazide and dialkyl acetylenedicarboxylates. The reaction conditions were optimized, obtaining an efficient, one-pot multicomponent reaction (Scheme 22).

Initially, the reactions were performed with a variety of solvents, such as methanol, ethanol 1,4-dioxane, acetonitrile and water, to understand the solvent’s effect on the yield. To optimize the better reaction conditions, various catalysts, such as KOH, triethylamine, piperidine, H_2_SO_4_, acetic acid and NaOH, were tested. It was observed that the ethanol is the best among the tested solvents in terms of yield and NaOH was identified as a suitable catalyst, which gave the maximum isolated yield.

The synthesized compounds were evaluated for their antiproliferative activity against the A549 lung cancer cell line and the antiproliferative activity of each individual compound was compared with Sorafenib, a Food and Drug Administration (FDA) approved drug standard. Among all the tested compounds, **50** (IC_50_ = 0.930 μg/mL), **51** (IC_50_ = 0.808 μg/mL) and **52** (IC_50_ = 0.967 μg/mL) showed promising activity compared with the standard drug, sorafenib (IC_50_ = 3.779 μg/mL). From the antiproliferative activity data, it is evident that the presence of electron-rich species, such as the methoxy group, and electron withdrawing groups, such as fluoro and chloro on the pyrazoline ring, are fundamental for their antiproliferative activity. Furthermore, to gain a better understanding of the effectiveness of the ligand analogue molecules, they studied the interaction of their derivatives docked into the binding site of the epidermal growth factor receptor (EGFR) and vascular endothelial growth factor receptor type 2 (VEGFR2). The molecular docking studies indicated that compound **51** had the greatest affinity for the binding site of these receptors, and the docking results also give us a new direction to design new inhibitors.

**Scheme 23:**
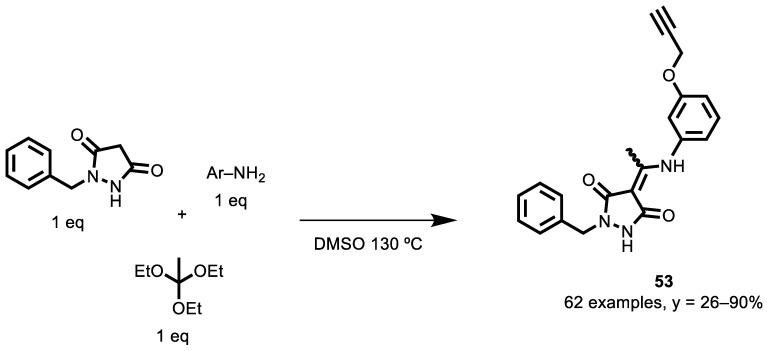
Synthetic route to prepare aza-analogs of TN-16.

To conclude this roundup of examples, the recent paper by Professor Tron’s group deserves special attention [146]. In this work, the authors developed a novel multicomponent reaction to prepare a library of 62 novel compounds, analogs of a potent antitubulin agent (TN-16), which are reported in Scheme 23. The synthesized compounds were evaluated for their possible antiproliferative activity on human neuroblastoma SH-SY5Y cells.

62 compounds were prepared via MCR between different anilines, triethylorthoacetate and 1-benzylpyrazolidine-3,5-dione, with a yield between 26 and 90%. When the compounds were screened on SH-SY5Y cells, only compound **52** (EC_50_ = 102 nM) showed significant cytotoxicity. These compounds were cytotoxic on cancer cell lines and, as expected from antitubulin agents, induce G2/M cell cycle arrest. In fact, these lead to a disruption of the microtubules and an increase in α-tubulin acetylation and affect in vitro polymerization, although they have a lesser effect in cellular tubulin polymerization assays.

## 4. Conclusions

Relevant, recent and representative papers dealing with the exploration of small molecule anticancer compounds assembled via MCRs have been highlighted, underlining the tremendous potential of MCRs in medicinal chemistry. An advantage of MCR chemistry is the ability to explore a very large chemical space for drug discovery and medicinal chemistry purposes. In view of the great need to synthesize compounds that enrich the arsenal against cancer, still largely an unmet clinical need, we hope that this review will be useful for the researchers, worldwide, interested in this field.

## Abbreviations

MCR (Multicomponent reaction), IMCRs (Isocyanide-based multicomponent reactions), NIMCRs (non-isocyanide-based multicomponent reactions), P-3CR (Passerini three component reaction), U-4CR (Ugi four component reaction), GBB-3CR (Groebke-Blackburn-Bienayme three component reaction), VL-3CR (Van Leusen three component reaction), O-3CR (Orru three component reaction), TosMIC (Tosylmethylisocyanide), BG-3CR (Biginelli three component reaction), G-3CR (Gewald three component reaction), PK-3CR (Pauson-Kand three component reaction), rhIDOI1(Human recombinant indoleamine 2,3-dioxygenase), MTT (3-(4,5-dimethylthiazol-2-yl)-2,5-diphenyl-2H-tetrazolium bromide), PA (Pyrazine-2-carboxylic acid), ROS (Reactive oxygen species), OA (Oleanolic Acid), MA (Maslinic Acid), SRB (Sulforhodamine B), DCM (Dichloromethane), MW (Microwave), SAR (Structure-activity relationship), mRNA (Messenger ribonucleic acid), EC_50_ (Half maximal effective concentration), MMP (Mitochondria membrane potential), EdU (5-ethynyl-2′-deoxyuridine), LPA (Lipophilic synthetic polyamines), FDA (Food and Drug administration), DHPM (dihydropyrimidinone), USI (Ultrasonic irradiation), EGFR (Epidermal growth factor receptor), VEGFR2 (vascular endothelial growth factor receptor type 2).
